# A dynamic i-motif with a duplex stem-loop in the long terminal repeat promoter of the HIV-1 proviral genome modulates viral transcription

**DOI:** 10.1093/nar/gkz937

**Published:** 2019-10-29

**Authors:** Emanuela Ruggiero, Sara Lago, Primož Šket, Matteo Nadai, Ilaria Frasson, Janez Plavec, Sara N Richter

**Affiliations:** 1 Department of Molecular Medicine, University of Padua, 35121 Padua, Italy; 2 Slovenian NMR center, National Institute of Chemistry, Hajdrihova, 19, Ljubljana SI-1000, Slovenia

## Abstract

I-motifs are non-canonical nucleic acids structures characterized by intercalated H-bonds between hemi-protonated cytosines. Evidence on the involvement of i-motif structures in the regulation of cellular processes in human cells has been consistently growing in the recent years. However, i-motifs within non-human genomes have never been investigated. Here, we report the characterization of i-motifs within the long terminal repeat (LTR) promoter of the HIV-1 proviral genome. Biophysical and biochemical analysis revealed formation of a predominant i-motif with an unprecedented loop composition. One-dimensional nuclear magnetic resonance investigation demonstrated formation of three G-C H-bonds in the long loop, which likely improve the structure overall stability. Pull-down experiments combined with mass spectrometry and protein crosslinking analysis showed that the LTR i-motif is recognized by the cellular protein hnRNP K, which induced folding at physiological conditions. In addition, hnRNP K silencing resulted in an increased LTR promoter activity, confirming the ability of the protein to stabilize the i-motif-forming sequence, which in turn regulates the LTR-mediated HIV-1 transcription. These findings provide new insights into the complexity of the HIV-1 virus and lay the basis for innovative antiviral drug design, based on the possibility to selectively recognize and target the HIV-1 LTR i-motif.

## INTRODUCTION

Nucleic acids can adopt several non-canonical secondary structures, such as triplexes, quadruplexes and hairpins, which are generally located within regulatory genomic regions and consequently involved in the regulation and function of genes. Quadruplexes, which are characterized by four single strands held together through non-Watson-Crick hydrogen (H)-bonds, can form both in guanine (G)- and cytosine (C)-rich strands. In the first case, four G residues are linked through Hoogsteen H-bonds to form a tetrad, and two or more tetrads self-stack to form the so-called G-quadruplex (G4) (1). G4s have been identified in crucial regulatory regions of the human genome, namely telomeres (2), DNA replication origins and oncogene promoters (3) where they are involved in the regulation of transcription (4).

**Figure 1.**
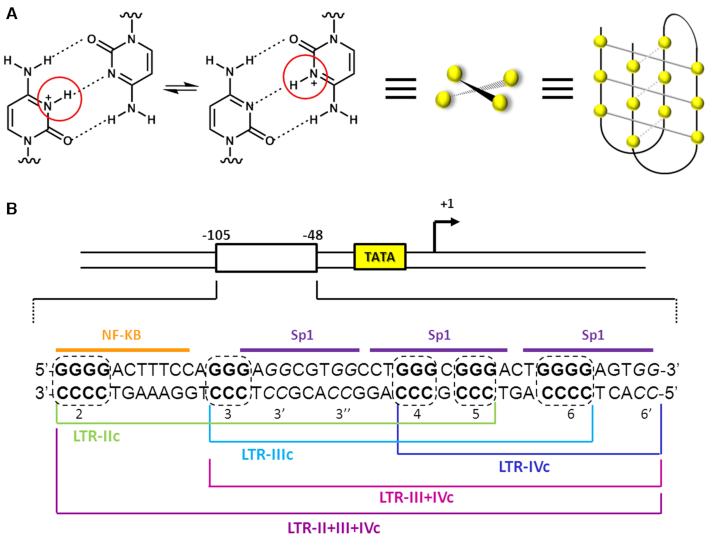
I-motifs in the HIV-1 LTR. (**A**) Hemiprotonated C^+^-C base pair: the N-3 of two Cs share a single proton (46). Two or more base pairs intercalate to form the folded i-motif structure. (**B**) The sequence of HIV-1 LTR U3 integrated region is reported: dashed lines comprise GC-boxes located within the G-rich and C-rich strands, which are consecutively numbered.

The strand complementary to G4s is C-rich and here C-quadruplex or i-motif structures can form (5). I-motifs fold thanks to intercalated H-bonds, which take place between hemi-protonated Cs (C^+^-C), forming two double-stranded filaments linked in an anti-parallel way (Figure 1A). So far, the i-motif is the only known DNA secondary structure which employs an intercalated system (6). When compared to B-DNA, i-motifs are characterized by a closer base pair distance, with two wide and two very narrow grooves: the consequent spatial arrangement favors sugar-sugar Van der Waals interactions which highly contribute to the stability of the structure. The presence of 2′-OH groups in the RNA sugar limits this connection, reducing the stability of i-motifs within RNA nucleic acids (7,8). The three-dimensional organization of C^+^-C base pairs allows formation of two intercalated topology, 3′-E and 5′-E, according to the position of the terminal C^+^-C base pair, which can form at the 3′-end or 5′-end, respectively. These two conformations differ for the interaction of the phosphodiester backbones through the narrow groove: in the 3′-E topology the sugar pairs are closer, conferring greater stability to this conformation with respect to the 5′-E one (9).

Because of the required protonation, i-motifs are stabilized at slightly acidic pH levels, making them pH-sensitive DNA scaffolds. For this reason, they have been widely employed in nanotechnology as molecular switches, biosensors and nanomachines (10,11). Nonetheless, the i-motif stability can be influenced by a plethora of parameters, such as length and nature of the loops, length of C-tracts, cellular environment, epigenetic modifications, negative supercoiling and co-solvents (12,13).

The dynamic equilibrium of G4/i-motif arrangements in the human genome represents a key element in transcription, with mutual cooperation between the G- and the C-rich strands (14). To this end, interesting results have been obtained for some oncogene promoters, such as BCL-2, MYC and KRAS, where i-motifs have been demonstrated to actively participate in the transcriptional process. Indeed, in the BCL-2 promoter, stabilization of the i-motif through selective ligands resulted in a significant downregulation of the oncogene; on the other hand, the unwinding by means of the human ribonucleoprotein (hnRNP) L-like served as a switch-on of transcription (15–17). For the MYC proto-oncogene, antagonistic effects of nucleolin and hnRNP K proteins, which bind to the G4 and i-motif, respectively, were demonstrated (18). In the KRAS gene, in which the i-motif is in equilibrium with a hairpin species, Kaiser and co-workers revealed that the selective recognition of the i-motif in the Mid region by hnRNP K positively modulates KRAS transcription, while disruption of the i-motif:protein complex downregulates gene expression (19). All these studies strongly support a role for i-motif DNA in transcription regulation and very recently the first direct visualization of i-motifs within the nuclei of human cells has been reported, through the employment of a specific antibody, iMab, thus providing a key turning point in the field of i-motif research (20).

While studies on G4-forming sequences have been extended also to microorganisms, such as yeasts (21), bacteria (22) and viruses (23–25), correlating G4s with several important human infectious diseases, i-motifs within non-human genomes have never been investigated. In this context, the long terminal repeat (LTR) promoter of the human immunodeficiency virus-1 (HIV-1) proviral genome has been demonstrated to be highly enriched in GC-content (Figure 1B), with the G-rich strand able to fold in different G4s, the stabilization of which downregulates viral transcription (26–28). The sequences forming G4s in the LTR overlap with transcription factors binding sites, in particular those for Sp1 and NFκB, feature that is highly conserved and shared by most primate lentiviruses (29). LTR G4s regulate the LTR-driven HIV-1 promoter activity through the interaction with cellular proteins, which can alternatively stabilize or unfold them (30,31); in addition, their targeting by G4-ligands results in strong antiviral activity (32,33).

Here we sought to investigate the characteristics of the C-rich strand in the HIV-1 LTR region, reporting for the first time the presence of i-motif structures within a viral genome. We showed through spectroscopic techniques that a major i-motif structure can form within the integrated HIV-1 LTR and that its folding is kept in longer sequences which mimic a full-length condition. The bromine footprinting protection assay and preliminary nuclear magnetic resonance (NMR) investigation provided the folding pattern of LTR i-motifs, revealing a polymorphic structure with an unprecedented loop composition. At last, we demonstrated that the LTR i-motif is recognized with high affinity by the cellular protein hnRNP K, which induces folding at physiological conditions and consequently modulates viral transcription.

## MATERIALS AND METHODS

### Oligonucleotides

All DNA oligonucleotides used in this work were purchased from Sigma-Aldrich (Milan, Italy) and are listed in Supplementary Table S1.

### Circular dichroism analysis

The oligonucleotides were diluted to final concentration (4 μM) in lithium cacodylate 10 mM buffer at different pH and KCl/NaCl/LiCl as indicated. All samples were folded by heating at 95°C for 5 min and subsequent cooling to room temperature 24 h before analysis. CD spectra were recorded on a Chirascan-Plus (Applied Photophysics, Leatherhead, UK) equipped with a Peltier temperature controller, using a quartz cell of 5 mm optical path length and an instrument scanning speed of 100 nm/min, with a response time of 4 s over a wavelength range of 230-320 nm. Each reported spectrum represents the average of 2 scans at 20°C and is baseline-corrected for signal contributions due to the buffer. Observed ellipticities were converted to mean residue ellipticity (*θ*) = deg × cm^2^ × dmol^−1^ (molar ellipticity). For the determination of melting temperatures (*T*_m_), spectra were recorded over a temperature range of 20-90°C (90-20°C for annealing experiments), with temperature increase of 5°C, followed by an equilibration step of 1 min. *T*_m_ values were calculated according to the van’t Hoff equation, applied for a two-state transition from folded to unfolded state, assuming that the heat capacity of the folded and unfolded states are equal.

### Br_2_-footprinting assay

The bromine footprinting assay protocol was adapted from a previously reported procedure (34). Briefly, gel-purified LTR-c oligonucleotides were 5′-end-labeled with [γ-^32^P] ATP by T4 polynucleotide kinase and purified by MicroSpin G-25 columns (GE Healthcare, Europe). They were next resuspended in lithium cacodylate 10 mM at given pH and KCl 100 mM, heat-denatured and folded. The oligomer 5 pmol was then incubated with molecular bromine formed *insitu* by mixing KBr with KHSO_5_ and the reaction was terminated by the addition of 0.3 M sodium acetate and calf thymus DNA solution. Unreacted bromine was removed by ethanol and tRNA precipitation steps. Subsequently, the DNA pellet was dried and resuspended in 100 mM piperidine solution. Samples were heated at 90°C for 30 min to induce bromination-specific strand cleavage, dried, washed and resuspended with sequencing gel loading dye. The cleavage was visualized on a 20% polyacrylamide sequencing gel with 7 M urea. The purine-specific reactions were performed using formic acid to generate sequencing markers (35). Gels were visualized by phosphorimaging (Typhoon FLA 9000, GE Healthcare Europe,Milan, Italy) and quantified by ImageQuant TL Software (GE Healthcare Europe, Milan, Italy).

### NMR spectroscopy

^1^H NMR spectra were collected on Agilent Technologies DD2 600 MHz NMR spectrometer at 298 and 273 K using a cold probe. DNA oligonucleotides were dissolved in H_2_O/D_2_O (9:1) solution containing KCl 100 mM and lithium cacodylate 10 mM, pH 5.0. Oligonucleotide concentrations were between 0.2 and 0.4 mM per strand. Double pulsed field gradient spin echo (DPFGSE) pulse sequence was used to suppress the water signal. Spectra were processed with program VNMRJ (Agilent Technologies).

### Pull-down assay

93T449 (ATCC^®^ CRL-3043™) cells were grown in RPMI (Gibco, Thermo Fisher Scientific, Waltham, MA, USA) supplemented with 10% heat-inactivated FBS serum (Gibco, Thermo Fisher Scientific, Waltham, MA, USA). Protein nuclear extracts were obtained by using NX-TRACT kit (Sigma-Aldrich, Milan, Italy) and quantified using Pierce™ BCA Protein Assay Kit (Thermo Fisher Scientific, Monza, Italy).

Biotinylated oligonucleotides 150 pmols were folded in phosphate buffer pH 7.4 20 mM, supplemented with KCl 100 mM, and bound to 50 μl of streptavidin-coated magnetic beads (Dynabeads^®^ M-280 Streptavidin, Thermo Fisher Scientific, Monza, Italy). DNA-coupled beads were then incubated with nuclear extracts 25 μg at 4°C for 2-3 h and protein excess was washed. Proteins were eluted first in NaCl 2M and then in 2× Laemmli buffer (SDS 4%, glycerol 80%, Tris-HCl pH 6.8 120 mM, 1,4-Dithiothreitol (DTT) 200 mM, bromophenol blue 0.02%), separated on a sodium dodecyl sulphate-polyacrylamide gelelectrophoresis (SDS-PAGE), stained with colloidal Coomassie staining (CBB G-250 0.02% w/v, aluminum sulfate-(14-18)-hydrate 5% w/v, ethanol 10% v/v, orthophosphoric acid 2% v/v) and cut in fractions corresponding to different molecular weights ranges. Bands were treated according to an established in-gel digestion protocol, as previously described (30). The obtained peptide mixture was finally concentrated in SpeedVac (Hetovac VR-1, Heto Lab Equipment, Denmark) to 1 μl and resuspended in 30 μl of formic acid 1%. The peptide mixture was analyzed by LC-MS. The injection was automatically performed by a low pressure Acquity H-class bioQuaternary Solvent Manager UPLC (Waters, Manchester, UK) system and a Jupiter proteo^®^ RP12 (1.0 × 150 mm, 4 μm, 90Å) (Phenomenex, Torrance, CA, US) chromatographic column. The detection was performed with a mass analyzer Xevo G2-XS QTof mass spectrometer (Waters, Manchester, UK). Instrument control, data acquisition and data processing were performed with MassLynx 4.1 software (Waters Corp.). Parent ions having the charge state 4^+^, 3^+^ and 2^+^ signals more intense than 200 counts and the related MS fragments ion signals more intense than 50 counts were employed to perform a Mascot Database Search to identify their parent protein. The matched proteins were deemed positively identified when two or more peptides provided a Mascot score >20- and 2-fold higher than the controls.

### I-motif-binding proteins crosslinking assay

Protein nuclear extracts were obtained as described in the pull-down experiment. Biotinylated oligonucleotides 150 pmol, folded in phosphate buffer pH 7.4 100 mM and KCl 100 mM, were bound to 50 μl of streptavidin-coated magnetic beads. DNA coupled-beads were incubated with nuclear proteins 150 μg extract at 4°C for 90 min and proteins excess was washed with Tris-HCl pH 7.5 50 mM-NaCl 150 mM solution. Samples were fixed with formaldehyde (Sigma-Aldrich, Milan, Italy) 5% for 30 min at room temperature, washed and then analyzed by western blot analysis, with an anti-hnRNP K antibody (mouse monoclonal D-6; Santa Cruz Biotechnology, Dallas, TX, USA). Briefly, samples were electrophoresed on a 10% SDS-PAGE and transferred to a nitrocellulose blotting membrane (Amersham TM Protan TM, GE Healtcare Life science, Milan, Italy) by using trans-blot SD semi-dry transfer cell (Bio-Rad Laboratories, Milan, Italy). The membrane was blocked with 2.5% skim milk in phosphate-buffered saline solution, incubated with the anti-hnRNP K antibody and then with the ECL Plex Goat-α-Mouse IgG-Cy5 (GE Healthcare Life sciences, Milan, Italy). At last, images were captured on Typhoon FLA 9000.

### FRET and FRET-melting assays

For Föster Resonance Energy Transfer (FRET) experiments, oligonucleotides were diluted to 0.1 μM in phosphate buffer pH 7.4 20 mM, or lithium cacodylate pH 7.4 10 and KCl 100 mM, heat denatured for 5 min at 95°C, and allowed to cool to room temperature for 16 h. Samples were incubated alone or with recombinant hnRNP K 500 ng (OriGene Technologies, Rockville, MD, USA) for 1 h at 4°C and subsequently fluorescence intensity was measured in a LightCycler II (Roche, Milan, Italy) by observing 6-carboxyfluorescein (*6*FAM) emission. The excitation wavelength was set at 480 nm and the emission was recorded from 500 to 650 nm. In the melting assay, fluorescence was monitored from 30°C to 95°C (1°C/min).

### Electrophoretic mobility shift assay

Electrophoretic mobility shift assay (EMSA) was performed as previously described (36). Briefly, radiolabeled LTR-IIIc (5 pmol) was resuspended in lithium cacodylate buffer (pH 7.4 10 mM, KCl 100 mM), heat-denatured and folded at room temperature. The binding reactions with hnRNP K (0-700 ng) were performed in binding buffer (Tris-HCl 20 mM pH 8, KCl 30 mM, MgCl_2_ 1.5 nM, DTT 1 mM, glycerol 8%, protease inhibitor cocktail 1%, NaF 5mM, Na_3_VO_4_ 1 mM) for 1 h in ice bath. Samples were run in 8% non-denaturing polyacrylamide gels for 17 h at 40 V, dried and visualized by phosphorimaging (Typhoon FLA 9000, GE Healthcare Europe, Milan, Italy). The equilibrium dissociation constant (*K*_D_) was determined by quantitating the radiolabeled signal in each sample and subtracting background, using ImageQuant TL software (GE Healthcare Europe, Milan, Italy). The bound DNA fraction was determined from the background-subtracted signal intensities using: bound/(bound+unbound). The DNA bound fraction was then plotted versus the hnRNP K concentration and data were fitted applying the following binding equation to perform non-linear regression, using SigmaPlot (Systat Software, San Jose, CA): f = Bmax*abs(x)/(*K*_D_ + abs(x)).

### siRNA and luciferase reporter assay

Gene-specific pooled siRNA trilencer targeting human hnRNP K and a negative control duplex were purchased from Origene (NCL Trilencer-27 Human siRNA, OriGene Technologies, Rockville, MD, USA). Human embryonic kidney (HEK) 293T cells (ATCC^®^ CRL-3216) were grown in DMEM (Gibco, Thermo Fisher Scientific, Waltham, MA, USA) supplemented with 10% heat-inactivated fetal bovine serum (FBS, Gibco, Thermo Fisher Scientific, Waltham, MA, USA). Cells were transfected with hnRNPK siRNA 50 nM or control siRNA by using Lipofectamine RNAiMAX (Invitrogen, Thermo Fisher Scientific, Waltham,MA, USA) following the manufacturer's instructions. pLTR luciferase plasmid (pGL4.10-LTR) (26) was transfected into the same cells 24 h post-silencing by using Lipofectamine 3000 (Invitrogen, Thermo Fisher Scientific, Waltham, MA, USA). Firefly luciferase signal was measured using the britelite plus Reporter Gene Assay System (PerkinElmer Inc., Milan, Italy) at a Victor X2 multilabel plate reader (Perkin Elmer Italia, Milan, Italy). Signal was normalized to total protein content, determined by BCA assay which was performed after cell lysis and protein extraction. Silencing efficiency was ascertained by Western-blot analysis, as described above. Briefly, cell protein extracts was obtained by resuspending cells in RIPA Buffer (Tris-HCl pH 7.5 20 mM; NaCl 150 mM, EDTA 1 mM, NP-40 1%, sodium deoxycholate 1%, Na_3_VO_4_ 1 mM, protease inhibitors 1×). Protein content was quantified using the Pierce^®^ BCA Protein Assay Kit, electrophoresed in 10% SDS-PAGE and transferred to a nitrocellulose blotting membrane. Blotting and visualization of membranes was performed as reported above.

## RESULTS

### The HIV-1 LTR C-rich sequence folds into i-motif structures

**Figure 2.**
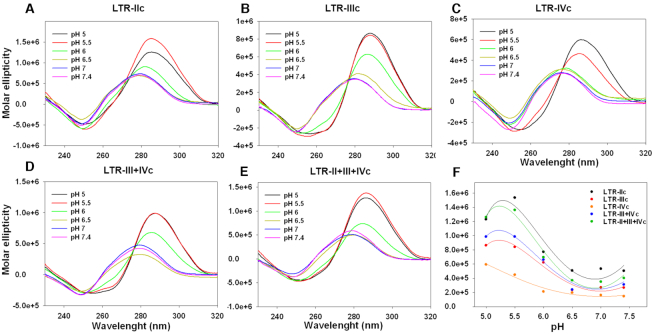
CD spectroscopy analysis of LTR-c sequences. Oligonucleotides (4 μM) were folded in lithium cacodylate 10 mM at the indicated pH with KCl 100 mM. (**A**–**E**) CD spectra of all analyzed sequences performed at increasing pH levels. (**F**) pH transitions obtained by plotting the molar ellipticity as a function of pH.

The ability of the C-rich strand in the HIV-1 LTR promoter region to fold into i-motif secondary structures was assessed through a pH gradient circular dichroism (CD) analysis. The sequence includes two CCCC-tracts (runs 2 and 6), three CCC-tracts (runs 3, 4, 5) and three additional CC-islands (3′, 3″ and 6′) (Figure 1B). For this work, we selected three sequences, namely LTR-IIc, LTR-IIIc and LTR-IVc, each containing four C-tracts. The LTR-IIIc displayed the signature i-motif CD spectrum at acidic pH (pH ≤ 6), characterized by a positive and a negative peak around 287 and 260 nm, respectively (Figure 2B); this conformation was pH-dependent and was lost at pH ≥ 6.5. LTR-IIc (Figure 2A) and LTR-IVc (Figure 2C) showed i-motif-like CD patterns at pH ≤ 5.5 and the minimum peak was shifted toward lower wavelengths, suggesting formation of a weaker arrangement. It is worth noting that LTR-IIc includes two long loops (>8 nts) and LTR-IVc presents a bulged C-tract, which seemingly limit i-motif formation. We also added in our study two longer sequences, LTR-III+IVc and LTR-II+III+IVc, to investigate conditions closer to the full-length LTR sequence. Both sequences showed the pH-dependent i-motif signature (Figure 2D and E), with increased molar ellipticity with respect to the LTR-IIIc. The transitional pH (pH_T_) value, i.e. the pH at which the oligonucleotide is 50% folded, for the LTR-c sequences was quantitatively measured by plotting the maximum molar ellipticity versus the pH (Figure 2F): LTR-IIIc showed the highest pH_T_ (6.1), followed by LTR-III+IVc (5.9), LTR-IIc and LTR-II+III+IVc (both pH_T_ = 5.8) and LTR-IVc (5.7).

The effect of different salt types and concentrations on the folding of LTR-IIIc in a pH gradient was also investigated (Supplementary Figure S1). The oligonucleotide was folded in buffers containing 50, 100 or 150 mM of NaCl, KCl and LiCl. The nature and concentration of the employed counterion did not affect the folding pattern of the i-motif, leading to the same transitional pH (Supplementary Figure S2). These data are in line with those described in the literature (37), therefore we performed all the experiments using KCl 100 mM, as reported for the G4 counterpart (26).

**Figure 3.**
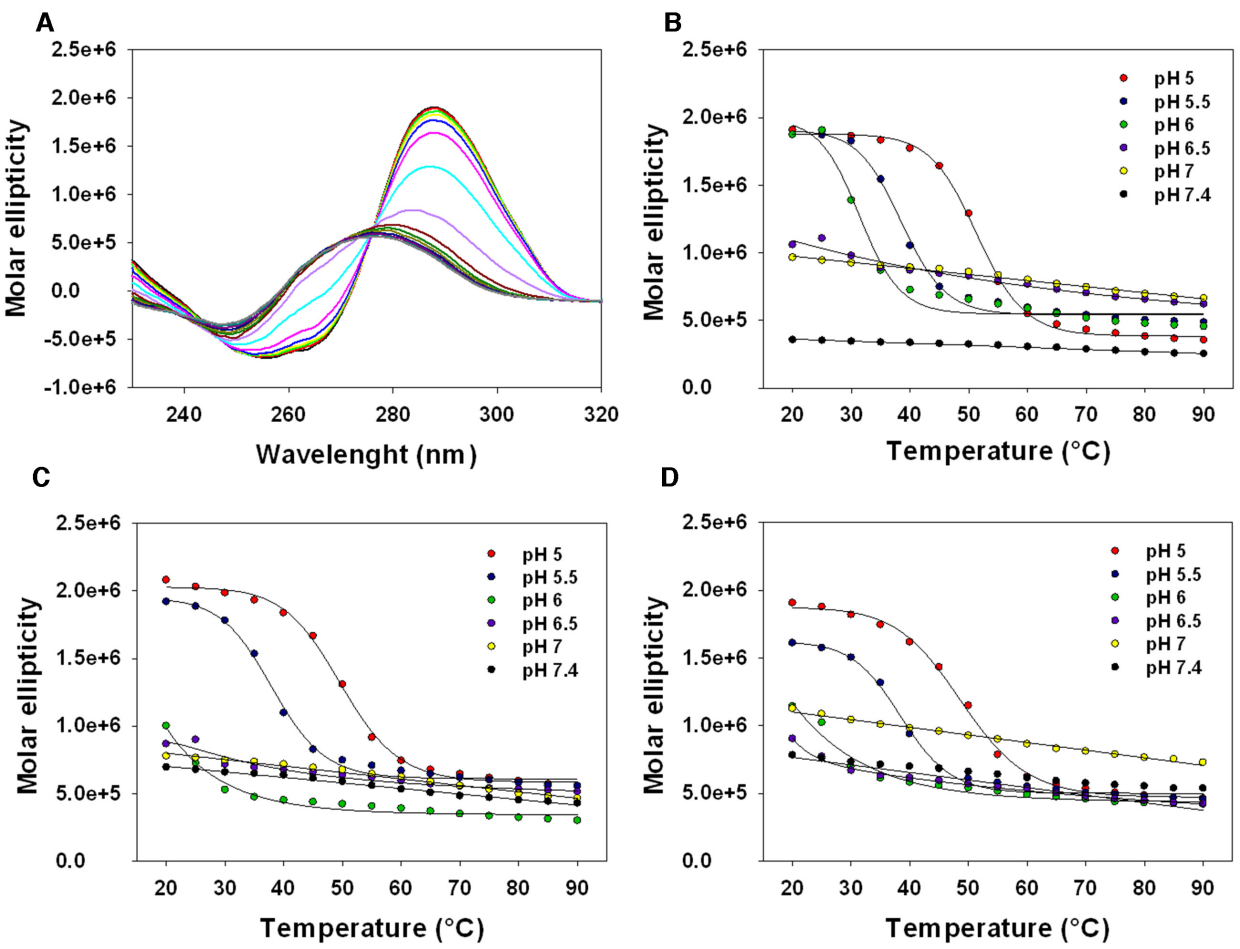
Denaturation profiles of LTR-c oligonucleotides. Oligonucleotides (4 μM) were folded in lithium cacodylate 10 mM at the indicated pH with KCl 100 mM. (**A**) CD thermal unfolding spectrum of LTR-IIIc performed at pH 5; spectra were recorded from 20 to 90°C. Molar ellipticity plotted versus temperature of LTR-IIIc (**B**), LTR-III+IVc (**C**) and LTR-II+III+IVc (**D**) at increasing pH values.

We next evaluated the thermal stability of the i-motif-forming LTR-c oligonucleotides by CD-melting experiments (Table 1). LTR-IIIc showed a *T*_m_ of 52.4°C at pH 5, which progressively decreased as the pH increased toward neutral levels (pH = 6.5), where the melting was no longer detectable (Figure 3 A and B; Supplementary Figure S3). The maximum peak resulted to be shifted to lower molar ellipticities as well as lower wavelengths with increasing temperature. At pH 5, LTR-III+IVc and LTR-II+III+IVc displayed *T*_m_ = 50.3°C and 48.9°C, respectively, comparable to that of LTR-IIIc (Table 1). However, for the longer sequences a folded-to-unfolded transition was measurable only up to pH 5.5, in accordance with the lower pH transition values (Figure 3C and D; Supplementary Figures S4 and 5).

**Table 1.** *T*_m_ of LTR-c sequences at increasing pH

	Tm (°C)^a^		
pH	LTR-IIIc	LTR-III+IVc	LTR-II+III+IVc
**5**	53.0 ± 0.3	50.3 ± 0.4	49.2 ± 0.5
**5.5**	42.8 ± 0.1	37.8 ± 0.2	38.4 ± 0.3
**6**	30.4 ± 0.1	ND^b^	ND^b^

^a^Data are reported as the mean of *n* = 2 experiments.

^b^ND = not detectable.

At pH 5, reannealing experiments were also performed, in which the molar ellipticity at *λ* = 288 nm was monitored from 90 to 20°C (Supplementary Figure S6). For all the three sequences, almost superimposable curves (melting and annealing) were observed, confirming a two-state transition with negligible hysteresis: these data indicate that the kinetics of association and dissociation of LTR-c oligonucleotides are very similar and corroborate the dynamic nature of the HIV-1 LTR i-motif.

### The HIV-1 LTR i-motif adopts a unique loop-folding pattern

**Figure 4.**
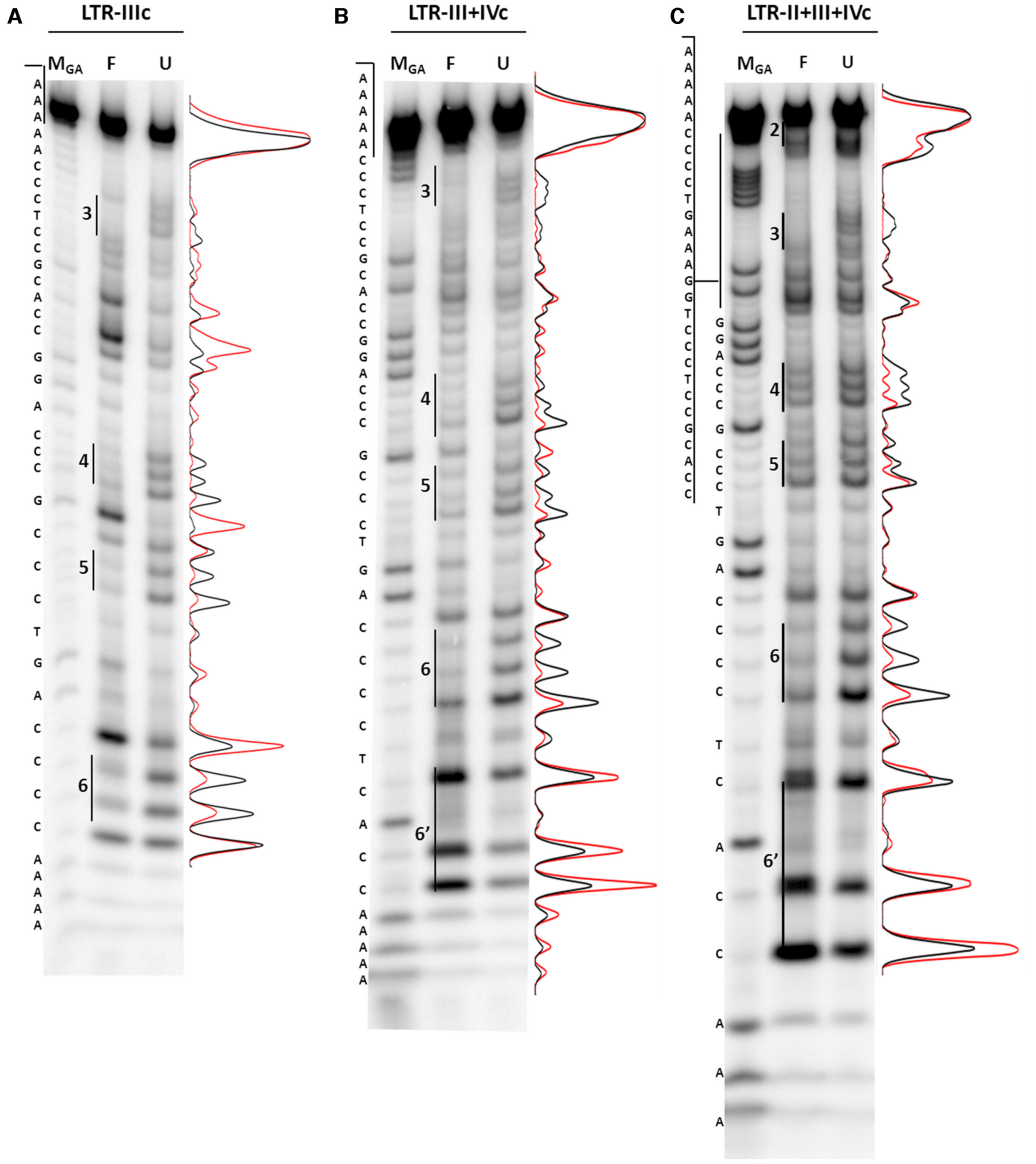
Br_2_-footprinting protection assay. LTR-IIIc (**A**), LTR-III+IVc (**B**) and LTR-II+III+IVc (**C**) oligonucleotides (5 pmol) were folded in lithium cacodylate 10 mM at pH 5 (F) or pH 7.4 (U), in the presence of KCl 100mM. M is a marker lane obtained with the Maxam and Gilbert sequencing protocol. Densitograms show quantification of cleaved bands intensity in the unfolded (black line) and folded (red line) conditions.

**Figure 5.**
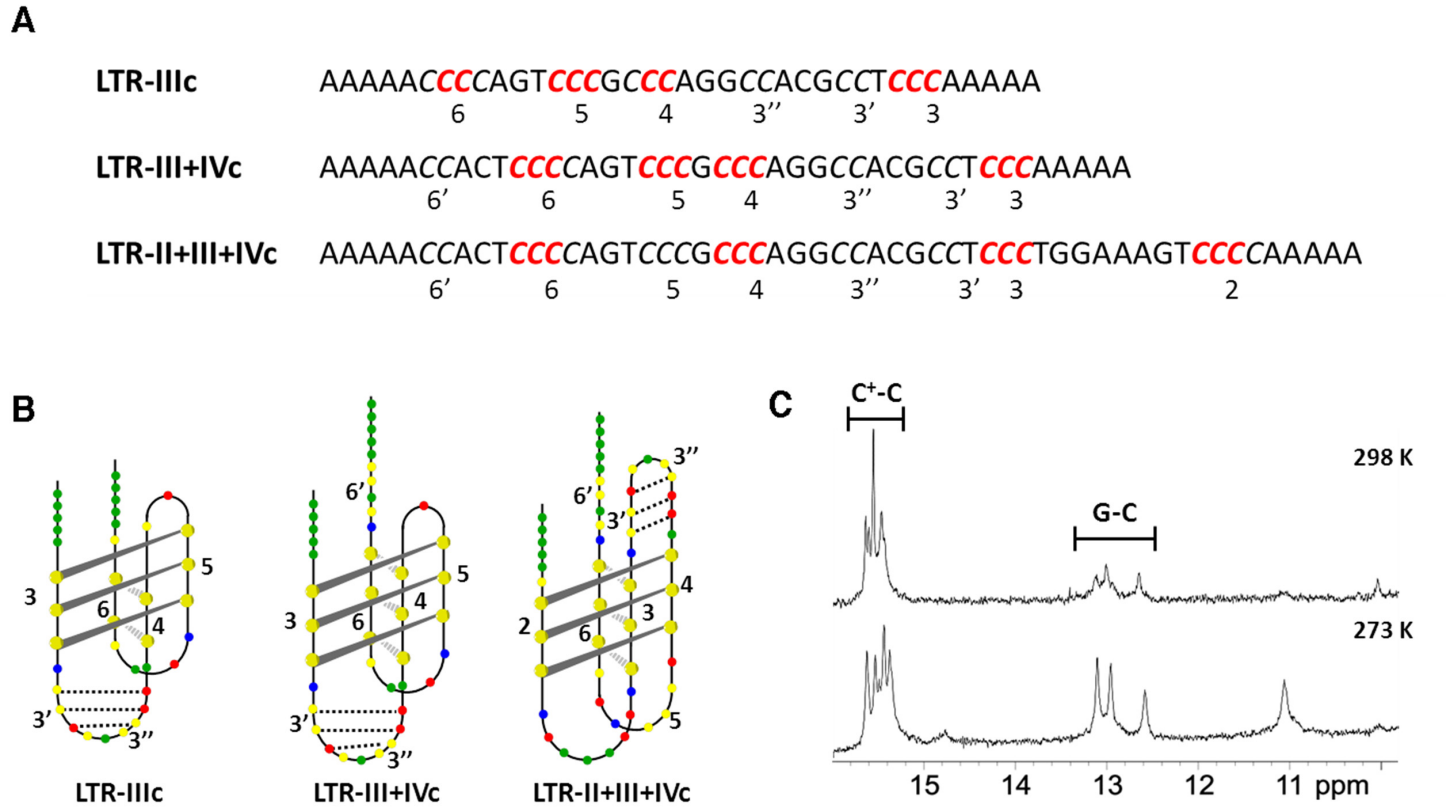
Structural analysis of LTR-c oligonucleotides. (**A**) Sequences of LTR-c oligonucleotides: C-tracts are reported in italic and numbered; the Cs involved in the i-motif formation are indicated in red. (**B**) Proposed models of LTR-c i-motif structures. G, T, C and A bases are shown in red, blue, yellow and green, respectively. Dotted lines represent G-C Watson-Crick H-bonds formed in the long loop. (**C**) Imino regions of ^1^H NMR spectra of LTR-IIIc at 298 K and 273 K in the presence of KCl 100 mM and lithium cacodylate 10 mM, pH 5. Spectra were recorded in 10% D_2_O. Concentration of oligonucleotide was 0.4 mM per strand. Chemical shift assignment to C^+^-C and G-C base pairing are provided.

To identify the Cs involved in the intercalated H-bonds of the LTR-c i-motif structures, we performed a bromine footprinting assay. Bromine is known to selectively react with C residues which are not involved in steric or electrostatic interactions, with preference for single stranded versus duplex DNA (34). As a consequence, only the free Cs in the loops, and not those involved in the C^+^-C base pairing, can be brominated and subjected to piperidine cleavage. Comparing the folded and unfolded conditions in the LTR-IIIc sequence (Figure 4A), the protection pattern revealed the four C-runs that are involved in i-motif formation. In detail, two consecutive Cs in runs 4 and 6 were protected from Br_2_, while three Cs were protected in repeats 3 and 5 (Figure 5A). Interestingly, the Cs adjacent to the protected ones were highly reactive to bromination, suggesting their location in the loops. In contrast, the unfolded oligonucleotide showed a uniform response to bromine reactivity, confirming its unstructured condition. These results suggest that the LTR-IIIc i-motif structure consists of three intercalated C^+^-C base pairs between C-runs 3 and 5, and two between C-runs 4 and 6, with a unique 4:2:11 loop folding pattern (Figure 5B). Protection assay performed on LTR-IIc and LTR-IVc (Supplementary Figure S7) confirmed the weaker arrangement suggested by the CD analysis, as only two C-islands were protected from bromination.

We next extended the footprinting analysis to the longer sequence LTR-III+IVc. This includes at its 3′-end an additional CCC-run (C-run 6′ in Figures 1B and 5A) interrupted by an adenine (A). In the G-rich sequence this tract is involved in the formation of a G4 with a bulge (38). The bromine footprinting assay revealed that LTR-III+IVc adopted a folding pattern quite similar to LTR-IIIc (lane F, Figure 4B), with the only difference of an additional C^+^-C base pair between C-runs 4 and 6, which leads to a 4:1:11 loops arrangement (Figure 5B). C-run 6′, which corresponds to the LTR-IVc region, is not involved in the folding of the LTR-III+IVc i-motif, showing that the i-motif that forms in the LTR-IIIc sequence is also the predominant form in the longer sequence. This evidence confirms the inability of LTR-IVc to fold, likely due to the presence of an interrupted (bulged) C-tract.

At last, we performed the bromine footprinting assay on the longest sequence, LTR-II+III+IVc, which most resembles the full-length C-rich LTR. Five out of the eight stretches of Cs present in the sequence resulted to be involved in the folding: since the intercalated conformation requires an even number of C-runs, likely more than one conformation can occur. Analyzing the densitograms, lower protection was observed in run 5 (Figure 4C), thus suggesting a major structure with a 7:11:8 loop folding arrangement (Figure 5B). As in LTR-III+IVc, also in this longest sequence six H-bonds were formed. The additional C^+^-C base pair in both LTR-III+IVc and LTR-II+III+IVc likely explains the increased ellipticity observed in the CD spectra. To note that, while the LTR-IIc segment itself does not fold into i-motif, its characterizing Cs participate in the formation of the i-motif in the longest sequence. According to the proposed models, all the HIV-1 LTR-c i-motif structures adopted the most stable 3′-E topology, as the outmost intercalated H-bond is located at the 3′-end (Figure 5B).

Overall, the footprinting analysis revealed the presence of a dynamic i-motif pattern, with the predominant structure formed within the LTR-IIIc segment. This is also the main region folded in G4 in the G-rich sequence (26).

### H-bonds patterns establish C^+^-C and G-C base pairs as integral units of LTR i-motif structures

The proposed folding topologies of LTR-c oligonucleotides (Figure 5B) allowed us to put up a hypothesis about the possible formation of additional intramolecular H-bonds in the long loop. Our group proved this possibility to occur within the LTR G-rich strand, where in-depth NMR analysis revealed that the LTR-III G4 is stabilized by the formation of three additional H-bonds in the long loop (39). Based on this evidence, we hypothesized that the same pattern could also take place in the LTR-IIIc, therefore NMR investigation was conducted to further assess LTR-c folding (Figure 5C and Supplementary Figure S8). LTR-c samples for NMR measurements were without 5′- and 3′-end overhangs involving five adenine residues, respectively.

Initially, 1D ^1^H NMR spectra were measured at 298 K and 273 K, at which signal-to-noise ratio of imino signals was improved. ^1^H NMR spectra of LTR-IIIc at 298 K and 273 K exhibited sharp signals between δ 15.2 and 15.8 ppm and between δ 12.5 and 13.2 ppm, which corresponded to imino protons of protonated Cs involved in C^+^-C base pairs and G residues in G-C base pairs, respectively (Figure 5C). The number of imino signals suggested that the structure was composed of six C^+^-C and three G-C base pairs, which is in agreement with the proposed folding topology, with an extra C^+^-C H-bond. At 273 K, additional signals at ca. δ 11 ppm were observed: these could be assigned to imino protons of thymine (T) residues.

For LTR-III+IVc, ^1^H NMR spectrum at 298 K revealed only a broad signal at ca. δ 15.5 ppm that is in agreement with formation of C^+^-C base pairs. Additional signals corresponding to imino protons of G residues involved in G-C base pairs as well as of imino protons of T residues appeared in the spectrum after lowering the sample temperature to 273 K. Despite their low intensity, three signals corresponding to imino protons of three G residues involved in G-C base pairs could be clearly resolved, in support of the proposed folding topology of LTR-III+IVc (Supplementary Figure S8).

^1^H NMR spectrum of LTR-II+III+IVc exhibited broad signals at 298 K indicating formation of C^+^-C and G-C base pairs as well as imino protons of T residues. In addition, a weak signal at ca. δ 14 ppm implied formation of A-T base pair within the structure of LTR-II+III+IVc. Integral ratio of imino signals corresponding to C^+^-C and G-C base pairs determined from ^1^H NMR spectrum at 273 K was about 6: 3, which supported the proposed folding topology of LTR-II+III+IVc (Supplementary Figure S8).

### The LTR i-motif is recognized and stabilized by the cellular protein hnRNP K

We previously demonstrated that the HIV-1 LTR region is modulated by two cellular proteins: nucleolin, which binds to and stabilizes the LTR G4s, silencing transcription (30) and the hnRNP A2B1, which unwinds the G4s, enhancing transcription (31). To investigate whether LTR-IIIc, the predominant i-motif, was also recognized by specific cellular proteins, we applied a combined pull-down/mass spectrometry (MS) approach. To this purpose, the biotinylated LTR-IIIc was linked to streptavidin-coated beads and incubated with nuclear proteins extracted from human cells. The bound proteins were then separated on SDS-PAGE gel, excised and subjected to MS analysis. A scrambled GC-rich sequence was used as control for non-specific protein binding. Interestingly, the most significant identified proteins were all part of the ribonucleoprotein family, i.e. hnRNP D, A2B1 and K, as listed in Table 2. However, whereas hnRNP D and A2B1 were also able to efficiently bind the control sequence, hnRNP K exhibited an impressive 7-fold selectivity for the LTR-IIIc sequence (Table 2). HnRNP K has already been reported to recognize and bind the i-motifs in the MYC and KRAS oncogene promoters, positively modulating their transcription. In both cases, protein binding occurs at the CT stretches located in the loops (40). Similarly, the LTR-IIIc long loop that includes a CCT element could be critical for hnRNP K recognition.

**Table 2.** Proteins identified by pull-down/MS analysis

	Score^a^	
Name	LTR-IIIc	Scrambled
hnRNP D	24	31
hnRNP A2B1	26	84
hnRNP K	127	17

^a^The score (assigned by mascot sofware, ‘http://www.matrixscience.com/help/interpretation _help.html#THRESH-OLDS’) (47) is the probability that the observed match is not a random event. The score is reported as −10x log10(P) where P is the absolute probability.

**Figure 6.**
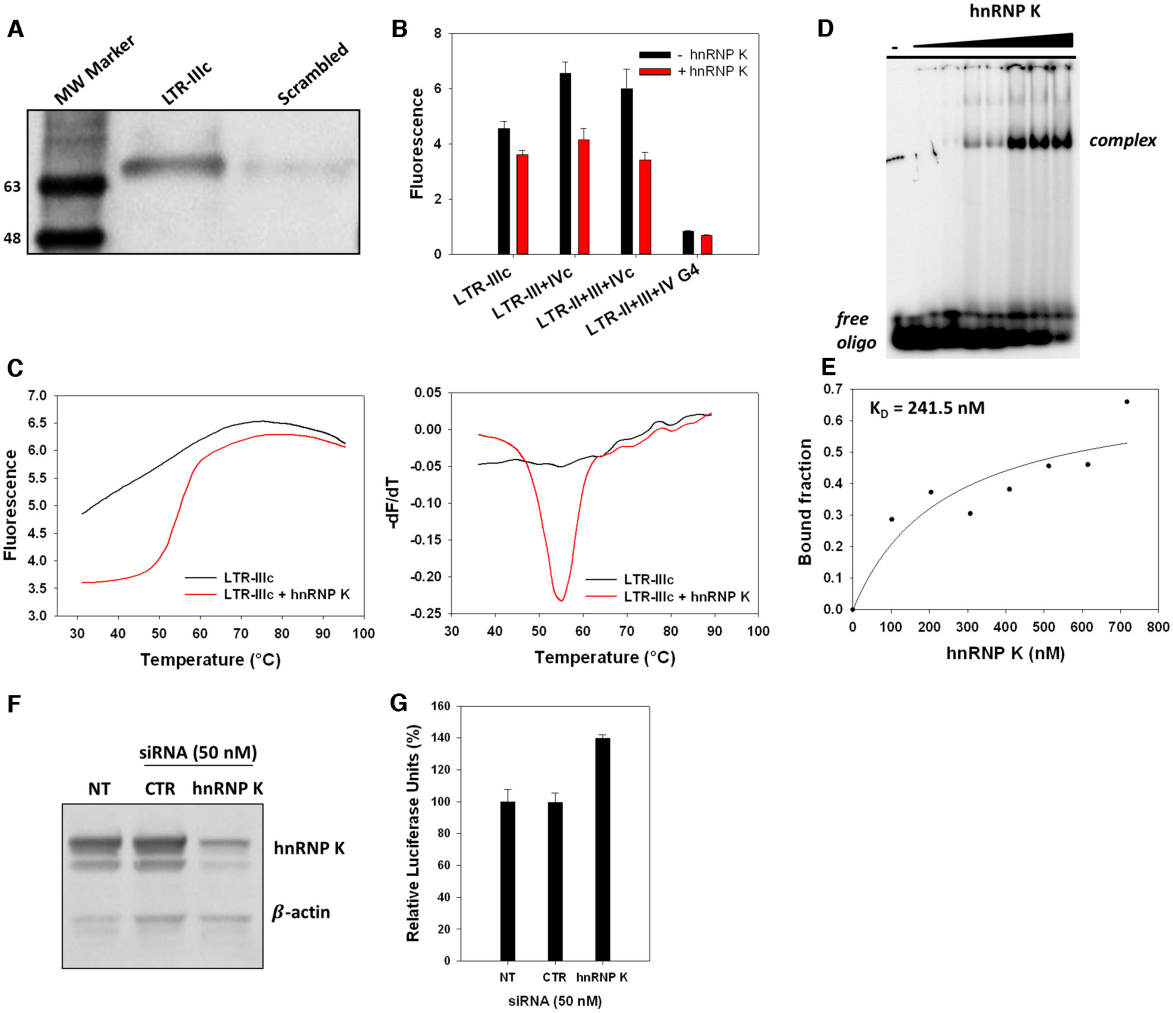
HnRNP K: LTR i-motifs binding interaction. (**A**) Western-blot analysis performed after i-motif-binding protein crosslinking, with the anti-hnRNP K antibody. (**B**) FRET assay on the LTR-c oligonucleotides (100 nM) at neutral pH (phosphate buffer pH 7.4 20 mM, KCl 100 mM), in the absence or presence of recombinant hnRNP K (500 ng). (**C**) FRET-melting assay of LTR-IIIc: melting curves (left panel) and corresponding first derivative curves (right panel) in the absence (black lines) and presence (red lines) of recombinant hnRNP K. (**D**) EMSA analysis of LTR-IIIc (5 pmol) performed at pH 7.4, in the absence and presence of hnRNP K (0–700 ng). (**E**) Plot of the bound fraction of LTR-IIIc versus increasing concentrations of hnRNP K. The *K*_D_ value was obtained by non-linear regression. (**F**) hnRNP K depletion in 293T cells by siRNAs, analyzed by western-blot with anti hnRNP K antibody. Detection of β-actin was used as control. NT indicates untreated cells and CTR indicates negative control siRNAs. (**G**) Analysis of the luciferase activity of the LTR promoter in 293T cells treated with hnRNP K siRNAs, normalized to protein content.

Concurrently, to further characterize the hnRNP K binding, the protein-DNA complex was subjected to crosslinking with formaldehyde and subsequent Western-blot, using a specific anti-hnRNP K antibody (Figure 6A): a clear band was observed for LTR-IIIc, while just a low signal was obtained for the control sequence, corroborating the preferential binding of hnRNP K for i-motif-forming sequences. We next conducted FRET experiments to examine the effect of hnRNP K on LTR i-motifs folding. Fluorophores-labeled LTR-c sequences, containing the FAM donor at the 5′-end and the TAMRA acceptor at the 3′-end, were incubated with recombinant hnRNP K for 1 h. The complementary LTR-II+III+IV sequence able to fold into G4 was used as control to monitor possible interactions with a different DNA secondary structure. As depicted in Figure 6B, in the presence of the protein an overall decrease in fluorescence was observed, suggesting that a strong FRET effect occurred between the fluorophores, which is to say that the two fluorophores were kept closer in the presence of the protein. No difference in the absence and presence of hnRNP K was shown on the G4 sequence, confirming the preferential binding of hnRNP K for i-motif-forming sequences. To further investigate this phenomenon, we moved to a FRET-melting system, monitoring fluorescence at increasing temperature. As shown in Figure 6C, at neutral pH LTR-IIIc was unstructured, according to previous CD analysis (Figure 2B); however when the protein was added, a clear sigmoidal curve, which indicates a complete folded-to-unfolded transition, was observed (Figure 6C). The same result was obtained with the LTR-III+IVc and LTR-II+III+IVc (Supplementary Figure S9A-B), proving that the hnRNP K stimulates the folding of LTR-c sequences into alternative structures at physiological conditions. No differences were observed for the G4 between the treated and untreated conditions (Supplementary Figure S9C). FRET assays were also carried out in lithium cacodylate buffer, which was employed in CD experiments, and yielded the same results obtained in phosphate buffer (Supplementary Figure S10), confirming that the hnRNP K structure-inducing effect is independent of any buffer contribution.

To further characterize the i-motif:hnRNP K binding affinity, EMSA was next performed at physiological conditions (pH 7.4). The LTR-IIIc oligonucleotide was analyzed in the absence and presence of increasing concentrations of protein (0-700 ng) (Figure 6D). Concentration-dependent formation of the DNA:protein complex was clearly visible. The *K*_D_ for this interaction, calculated through quantification of bands intensity, was estimated to be 241.5 nM (Figure 6E).

### Knockdown of hnRNPK increases viral transcription

To further investigate the biological role of the hnRNP K in the regulation of viral transcription, we evaluated the effect of hnRNP K depletion in a luciferase reporter system. Twenty-four hours after hnRNP K silencing, a luciferase reporter plasmid containing the HIV-1 LTR sequence (26) was transfected into cells and the luciferase signal was measured 24 h later. Knockdown of hnRNP K, which was confirmed through Western-blot analysis (Figure 6F), resulted in a strong increase in transcriptional activity, as reported in Figure 6G. This result corroborates the data collected in FRET experiments and strongly supports the ability of the hnRNP K to induce formation of i-motif structures, which act as downregulators of LTR transcription. Notably, this outcome is the opposite to that reported on c-MYC (18) and KRAS (19) oncogenes, where the hnRNP K binds to the i-motif structures to unfold them, thus promoting transcription.

## DISCUSSION

The LTR region within the HIV-1 proviral genome represents the control center for transcription of the HIV-1 genes and full-length genome. Its promoter activity lies in the U3 region where the binding sites for key cellular transcription factors, i.e. NF-κB and Sp1, are located. We previously demonstrated that the ability of this region to fold into G4s plays a key role in regulating viral transcription and that these G4s can be targeted to reduce virus propagation. With the purpose of better characterizing the HIV-1 LTR-mediated regulation, and ultimately to identify new targets for anti-HIV-1 therapy, we evaluated the presence of secondary structures within the C-rich strand in the LTR promoter. We showed that LTR-IIIc is the major HIV-1 LTR i-motif, the folding of which is dynamic and pH-dependent, in line with previously reported data on i-motifs in the human genome (6). It has been reported that viral infections vary the pH (pH_i_) of intracellular compartments (41) and, in particular, HIV-1 has been proved to reduce the pH_i_ in T-lymphoblastoids cells (42): thus we propose that LTR i-motifs are triggered during infection to modulate viral processes.

Targeting of these structures generally involves a specific recognition at loops levels (48), and is consequently strictly related to the i-motif topology. Bromine protection experiments provided detailed information on the LTR-IIIc i-motif organization, which includes four C-stretches separated by a unique 4:2:11 loop composition. Generally, long loops are detrimental to structure stability, unless they can form additional intramolecular interactions which contribute to the structure rigidity (43). Indeed, in the LTR-IIIc long loop, the primary sequence allows formation of additional Watson-Crick H-bonds, the actual occurrence of which was proven by one-dimensional ^1^H NMR spectroscopy. To our knowledge, this represents the first reported case of i-motif structure stabilized by multiple Watson-Crick H-bonds. Further in-depth analysis of this peculiar arrangement will help disclose a unique recognition site for the design of new ligands able to selectively target the HIV-1 LTR i-motif.

Intriguingly, when the longest LTR-II+III+IVc sequence that includes eight contiguous C-tracts was analyzed, the folding pattern was found to be rearranged, representing a 7:11:8 loops organization; the G-C H-bonds persisted also in the longer sequence, suggesting that targeting of the i-motif through recognition at the base-paired loop level represents a feasible approach, despite the dynamism of LTR-c i-motif.

We finally demonstrated that the LTR-IIIc is recognized and stabilized by the hnRNP K under physiological pH and ion composition. HnRNP K is a multifunctional protein, reported to be involved in the regulation of various cellular processes, like chromatin remodeling, transcription, RNA alternative splicing and translation (44). In the MYC (18) and KRAS (19) genes, hnRNP K is able to bind the i-motif structures located in the C-rich strands, stimulating their transcription: in both cases hnRNP K unfolds the i-motif structure, as demonstrated by FRET experiments. In contrast, we here reported that upon binding to LTR-IIIc detected in the pull-down and crosslinking experiments, the protein strongly stabilizes the folding. Indeed, the FRET-melting analysis revealed the induction of a folded structure at physiological pH, condition at which the sequence generally adopts a random-coil conformation. Stabilization of the i-motif structure by a cellular protein has never been observed so far; the BCL-2 oncogene i-motif has been reported to be stabilized by a cholestane-based compound which reduced the FRET signal by 50% (16), similarly to our data. However, such stabilization made the BCL-2 i-motif more susceptible to hnRNP LL recognition and subsequent unfolding. In our study, instead, depletion of hnRNP K enhanced LTR transcriptional activity, confirming the ability of i) the protein to induce folding; ii) i-motifs to stall the transcription machinery. If the observed hnRNP K-induced stabilization is defined also in other viral systems, it could represent a key element of discrimination between the virus machinery and its host, eventually making the hnRNP K: i-motif interaction a new target in antiviral therapy. Notably, hnRNP K has already been found to be involved in the regulation of HIV-1 transcription: hnRNP K has been characterized as a docking platform for several kinases, contributing, through a multimeric complex, to a strong Tat-dependent modulation of HIV-1 transcription (45). We suggest that one of the multiple processes in which hnRNP K is entangled, could involve the binding to the i-motif structure in the LTR promoter of the proviral genome.

**Figure 7.**
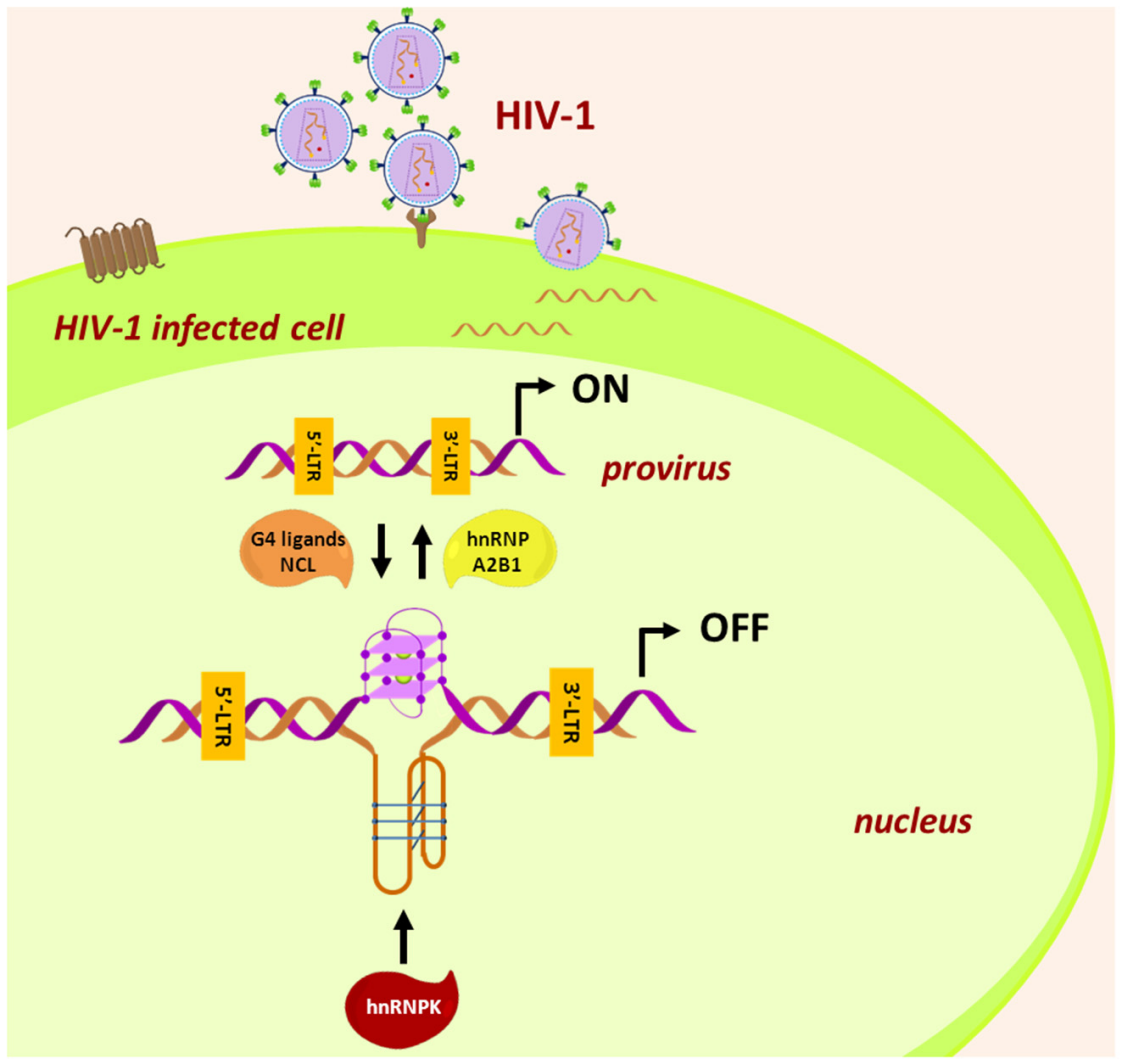
Schematic representation of the G4- and i-motif-mediated regulation at the HIV-1 LTR promoter.

Overall, this work provides new insights into the complexity of the HIV-1 virus, describing for the first time formation of i-motif structures within a non-human genome and their implication in viral transcription. The presence of i-motifs and G4s in the LTR promoter and their interaction with cellular proteins depict a finely tuned epigenetic regulation of HIV-1 transcription (Figure 7). Moreover, these outcomes lay the basis for innovative antiviral drug design, based on the possibility to selectively recognize the HIV-1 LTR i-motif.

## Supplementary Material

gkz937_Supplemental_File
