# Supplementary material for: A dynamic i-motif with a duplex stem-loop in the long terminal repeat promoter of the HIV-1 proviral genome modulates viral transcription
Source: Nucleic Acids Res. 2019 Oct 29;47(21):11057–68. doi: 10.1093/nar/gkz937 (PMC6868428; doi:10.1093/nar/gkz937)
Supplement: gkz937_Supplemental_File [file gkz937_supplemental_file.docx]

**Supporting Information**

**A dynamic i-motif with a duplex stem-loop in the long terminal repeat promoter of the HIV-1 proviral genome modulates viral transcription**

*Emanuela Ruggiero^1^, Sara Lago^1^, Primož Šket^2,^ , Matteo Nadai^1^, Ilaria Frasson^1^, Janez Plavec^2,^ and Sara N. Richter^1,*^*

^1^Department of Molecular Medicine, University of Padua, 35121 Padua, Italy

^2^Slovenian NMR center, National Institute of Chemistry, Hajdrihova, 19, Ljubljana SI-1000, Slovenia.

**Contents:**

Supplementary Table S1

Supplementary Figures S1-S10

**Table S1.** Oligonucleotides used in this study

| **Assay** | **Name** | **Sequence (5' - 3')** |
| --- | --- | --- |
| CD  Br_2_-footprinting  EMSA | LTR-IIc | AAAAACCCGCCCAGGCCACGCCTCCCTGGAAAGTCCCCAAAAA |
|  | LTR-IIIc | AAAAACCCCAGTCCCGCCCAGGCCACGCCTCCCAAAAA |
|  | LTR-IVc | AAAAACCACTCCCCAGTCCCGCCCAAAAA |
|  | LTR-III+IVc | AAAAACCACTCCCCAGTCCCGCCCAGGCCACGCCTCCCAAAAA |
|  | LTR-II+III+IVc | AAAAACCACTCCCCAGTCCCGCCCAGGCCACGCCTCCCTGGAAAGTCCCCAAAAA |
| ^1^H NMR | LTR-IIIc | CCCCAGTCCCGCCCAGGCCACGCCTCCC |
|  | LTR-III+IVc | CCACTCCCCAGTCCCGCCCAGGCCACGCCTCCC |
|  | LTR-II+III+IVc | CCACTCCCCAGTCCCGCCCAGGCCACGCCTCCCTGGAAAGTCCCC |
| Pull-down  Crosslinking | LTR-IIIc | (Btn)-AAAAACCCCAGTCCCGCCCAGGCCACGCCTCCCAAAAA |
|  | Scrambled | (Btn)-TTTTTGGAGTCGTGTCGCGTGTCGAGCGTGTGTAGTGGTTTTT |
| FRET | LTR-IIIc | (*6*FAM)-ACCCCAGTCCCGCCCAGGCCACGCCTCCCA-(TAMRA) |
|  | LTR-III+IVc | (*6*FAM)-ACCACTCCCCAGTCCCGCCCAGGCCACGCCTCCCA-(TAMRA) |
|  | LTR-II+III+IVc | (*6*FAM)-ACCACTCCCCAGTCCCGCCCAGGCCACGCCTCCCTGGAAAGTCCCCA-(TAMRA) |
|  | LTR-II+III+IV G4 | (*6*FAM)-TGGGGACTTTCCAGGGAGGCGTGGCCTGGGCGGGACTGGGGAGTGGT-(TAMRA) |


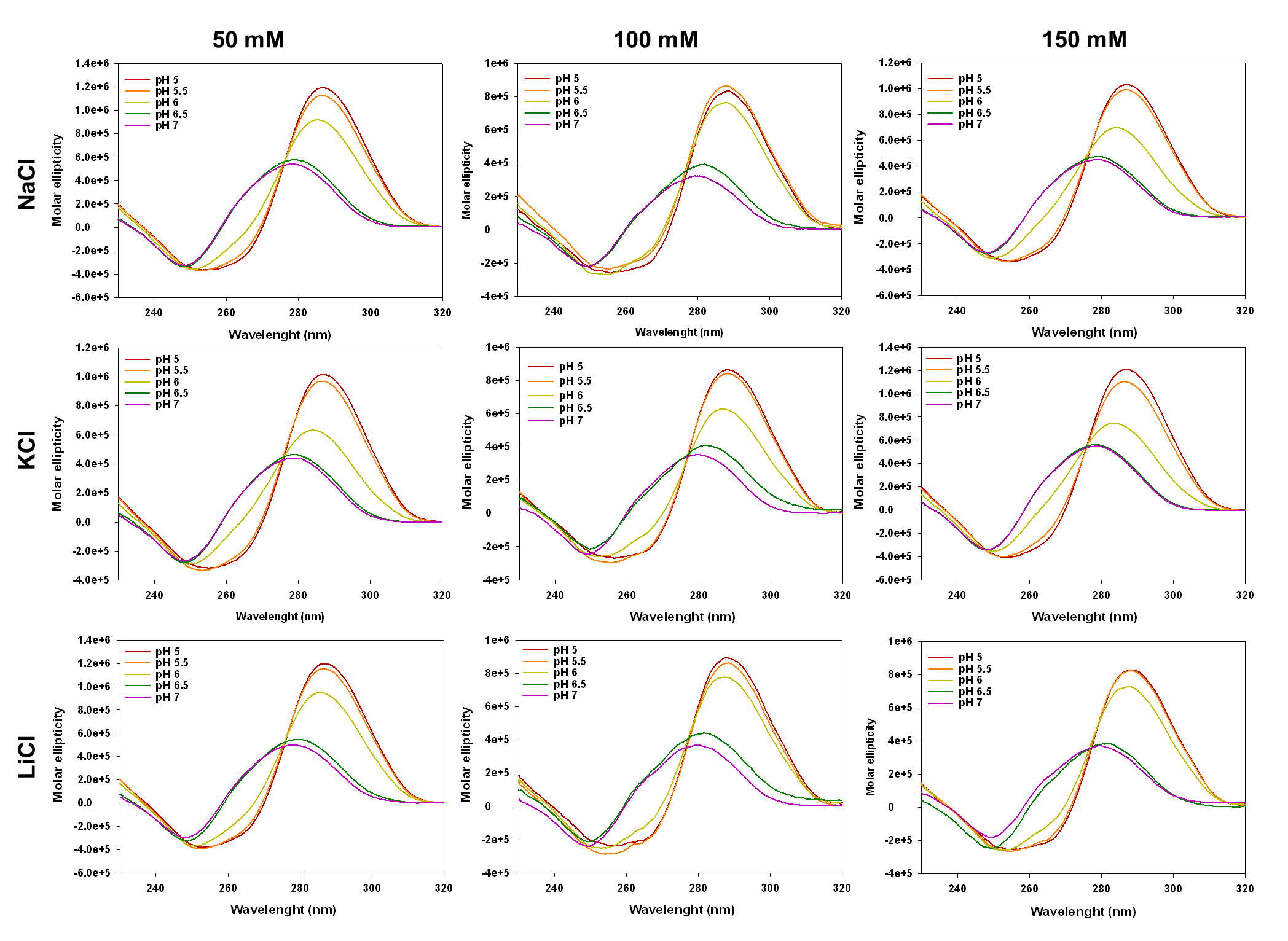


**Figure S1.** Effect of different salt types and concentrations on i-motif folding. CD curves of LTR-IIIc (4 µM) were measured at increasing pH levels, in the presence of sodium (upper panels), potassium (middle panels) and lithium (lower panels) chloride salts at different concentrations, as indicated.


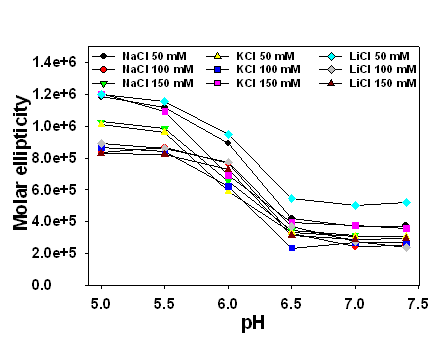


**Figure S2.** pH-transition curves in the presence of different salts. Curves were measured by plotting the molar ellipticity as a function of pH.


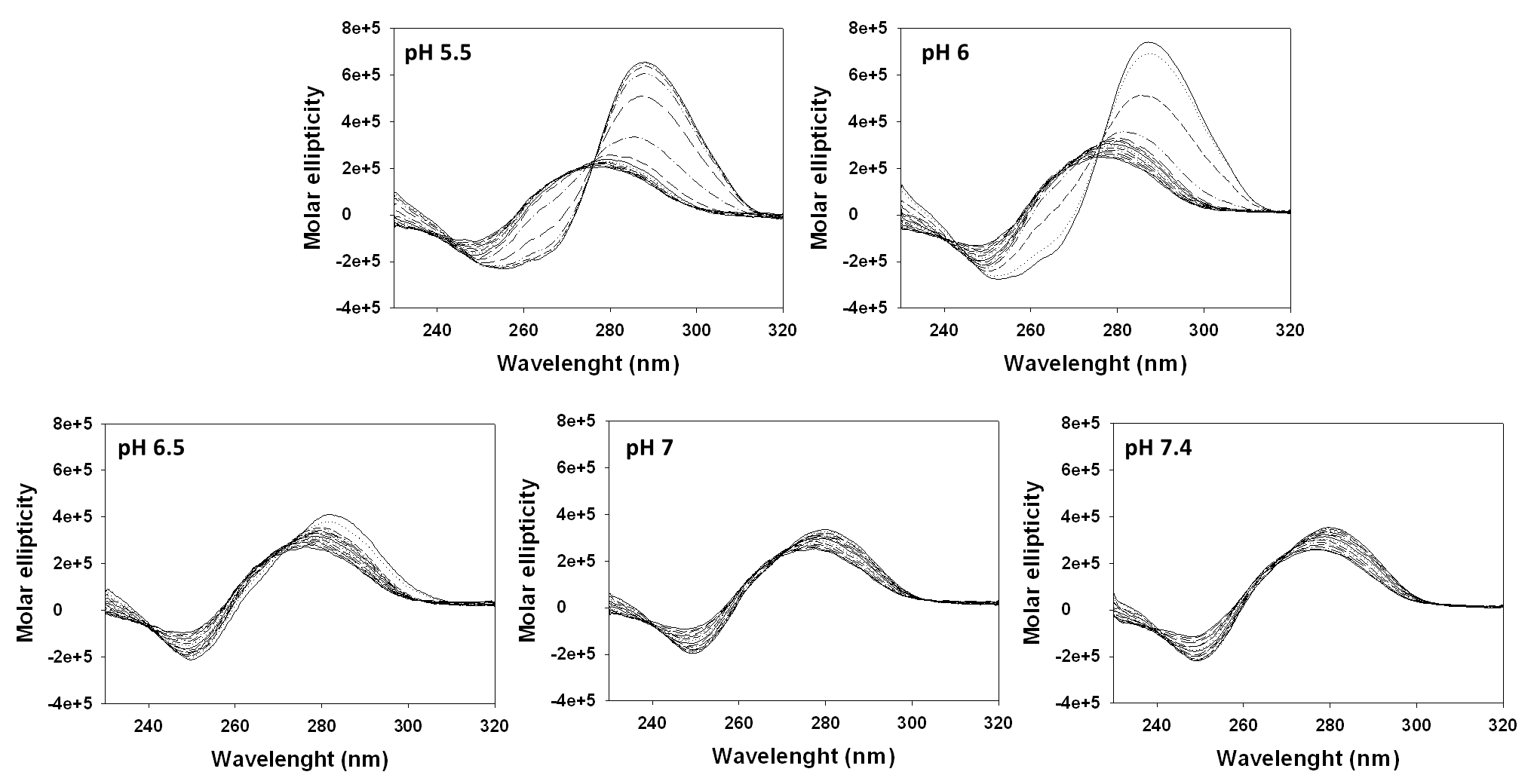


**Figure S3.** Denaturation profiles of the LTR-IIIc sequence. CD thermal unfolding spectra were measured at different pH conditions, as indicated.


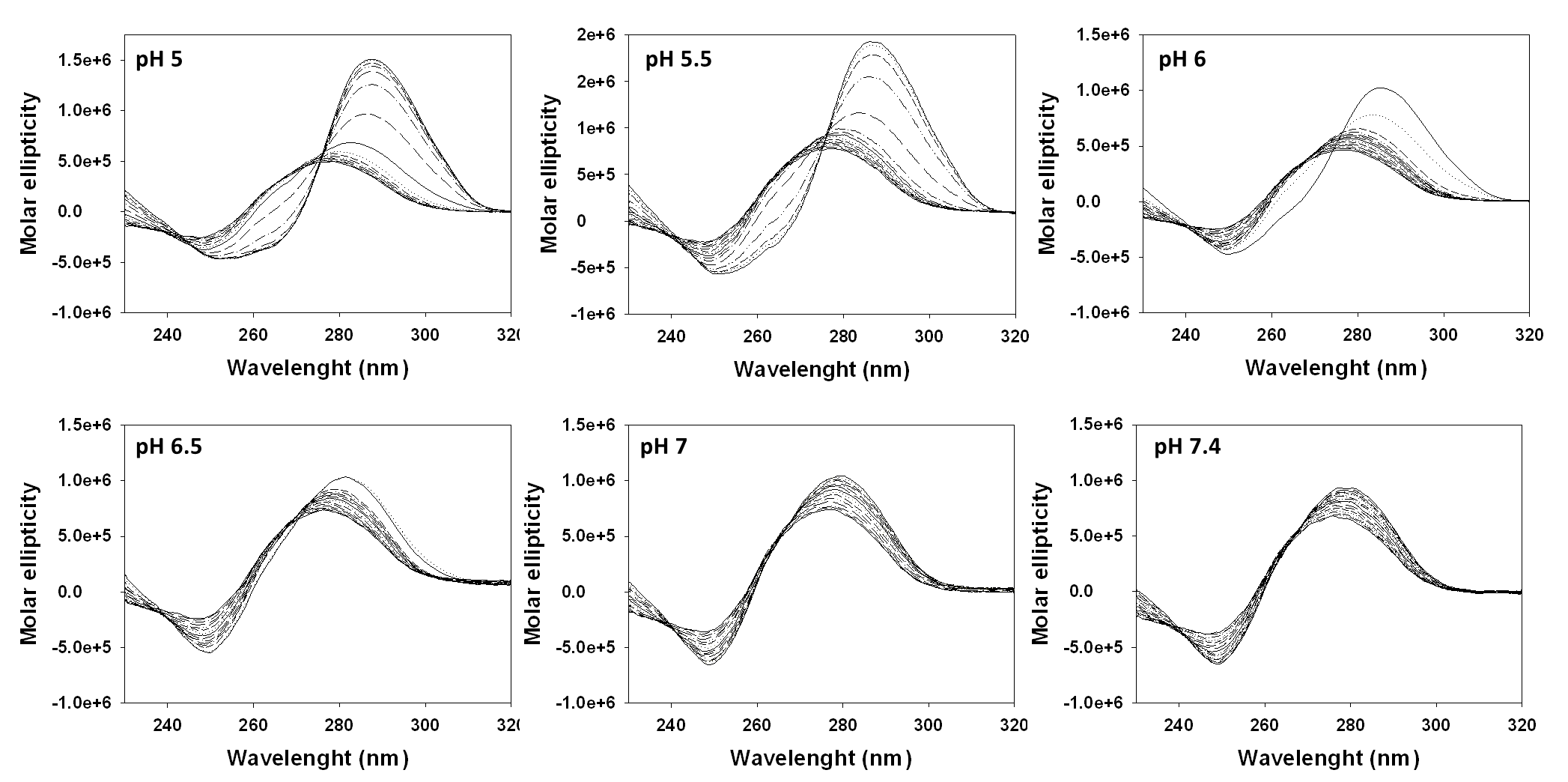


**Figure S4.** Denaturation profiles of the LTR-III+IVc sequence. CD thermal unfolding spectra were measured at different pH conditions, as indicated.


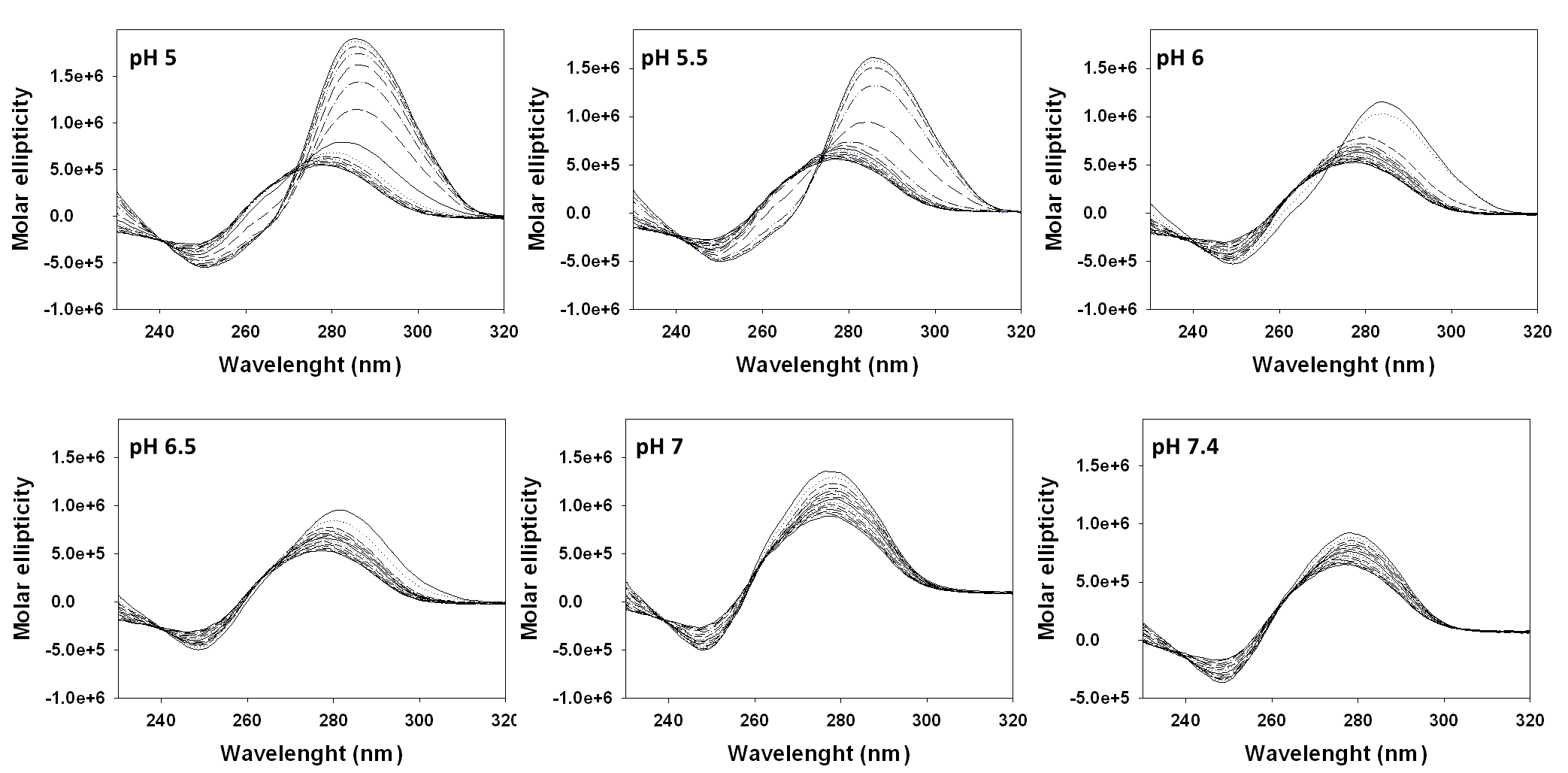


**Figure S5.** Denaturation profiles of the LTR-II+III+IVc sequence. CD thermal unfolding spectra were measured at different pH conditions, as indicated.


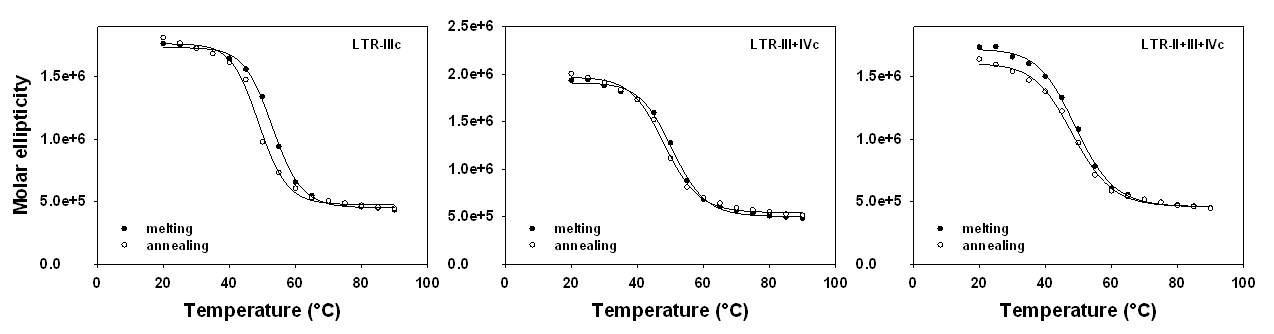


**Figure S6.** Melting-annealing profiles of LTR-c sequences at pH 5. The CD melting and annealing spectra were recorded after folding each oligonucleotide (4 µM) in lithium cacodylate 10 mM and potassium chloride 100 mM. Curves were obtained by monitoring the molar ellipticity at λ = 288 nm at increasing/decreasing temperature.

**
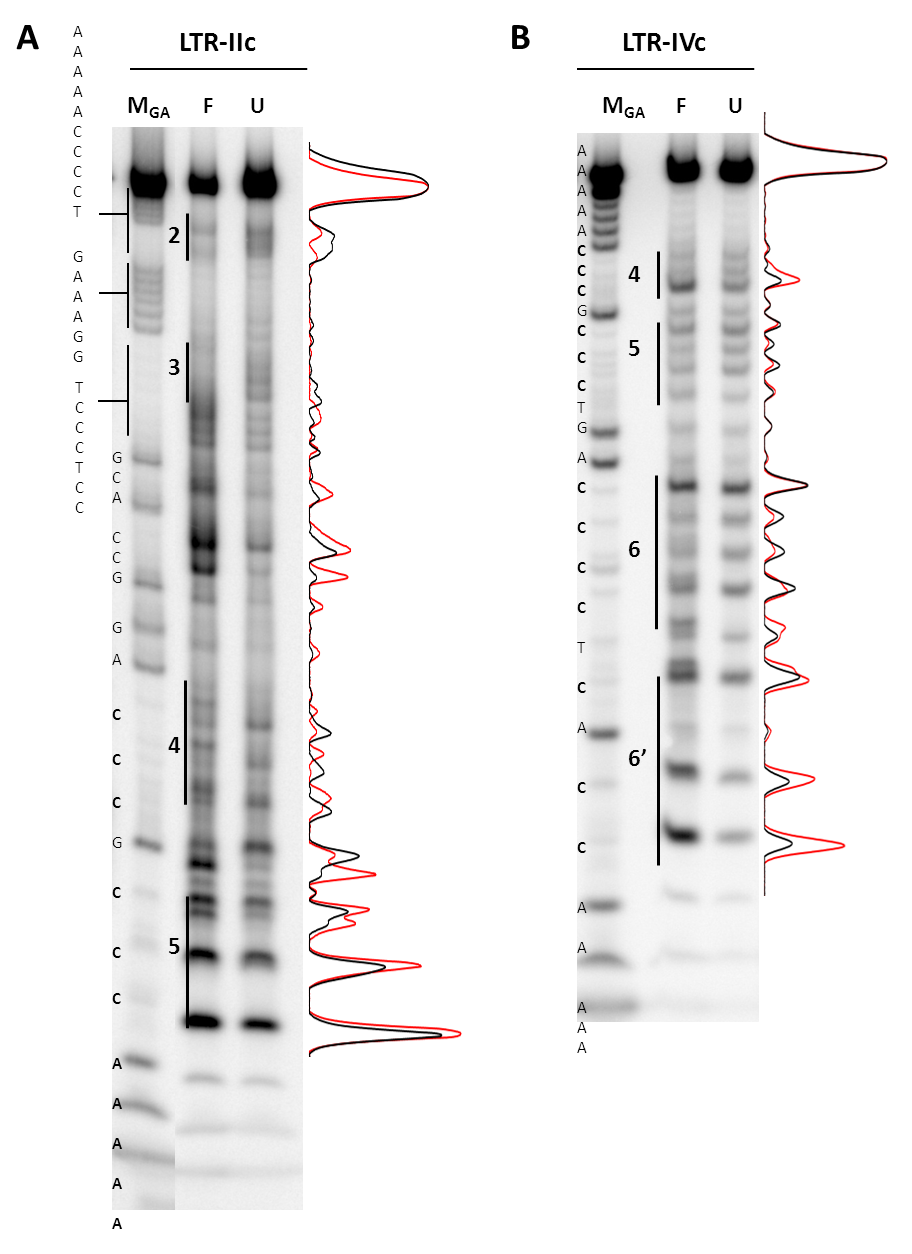
**

**Figure S7.** Br2-footprinting protection assay. LTR-IIc (A) and LTR-IVc (B) (5pmol) were folded at pH 5 (F) and pH 7.4 (U). M is a marker lane obtained with the Maxam and Gilbert sequencing protocol. Densitograms show quantification of cleaved bands intensity in the unfolded (black line) and folded (red line) conditions.


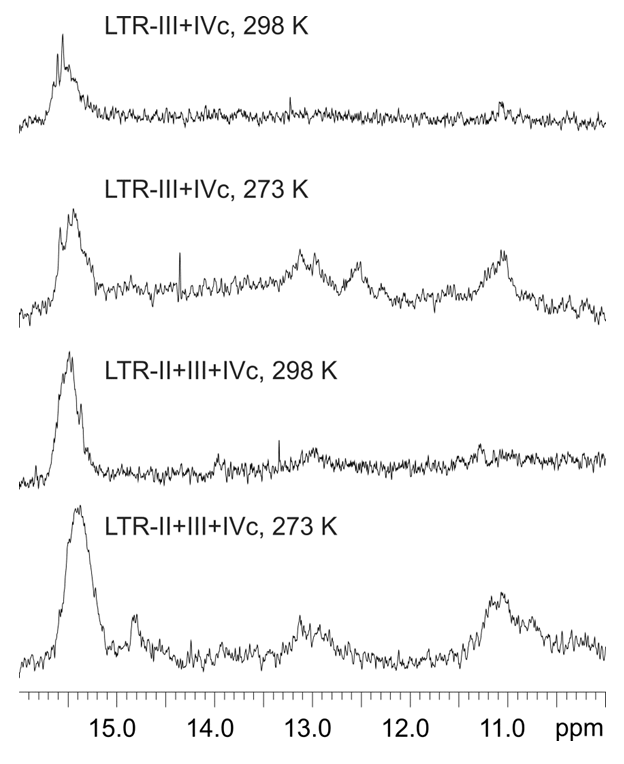


**Figure S8**. ^1^H NMR analysis. Imino regions of ^1^H NMR spectra of LTR-III+IVc and LTR-II+III+IVc sequences was performed at 298 and 273 K, in the presence of KCl 100 mM and lithium cacodylate 10 mM, pH 5. Spectra were recorded in 10 % D_2_O. Concentrations of oligonucleotides were between 0.2 and 0.4 mM per strand.


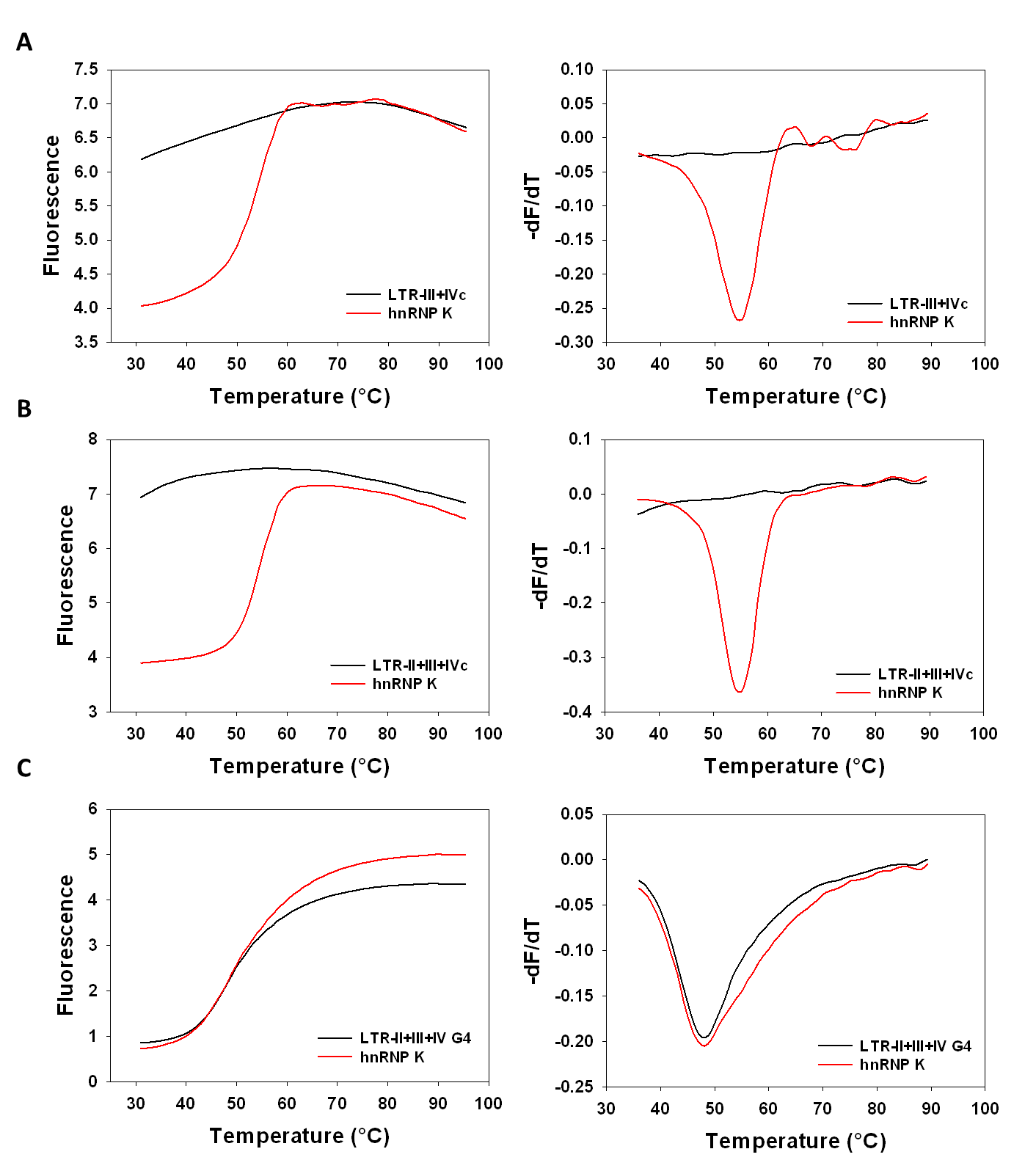


**Figure S9**. FRET-melting assay. Melting curves and corresponding first derivative curves of LTR-III+IVc (A), LTR-II+III+IVc (B) and LTR-II+III+IV G4 (C) (100 nM) in the absence (black lines) and presence (red lines) of recombinant hnRNP K (500 ng).


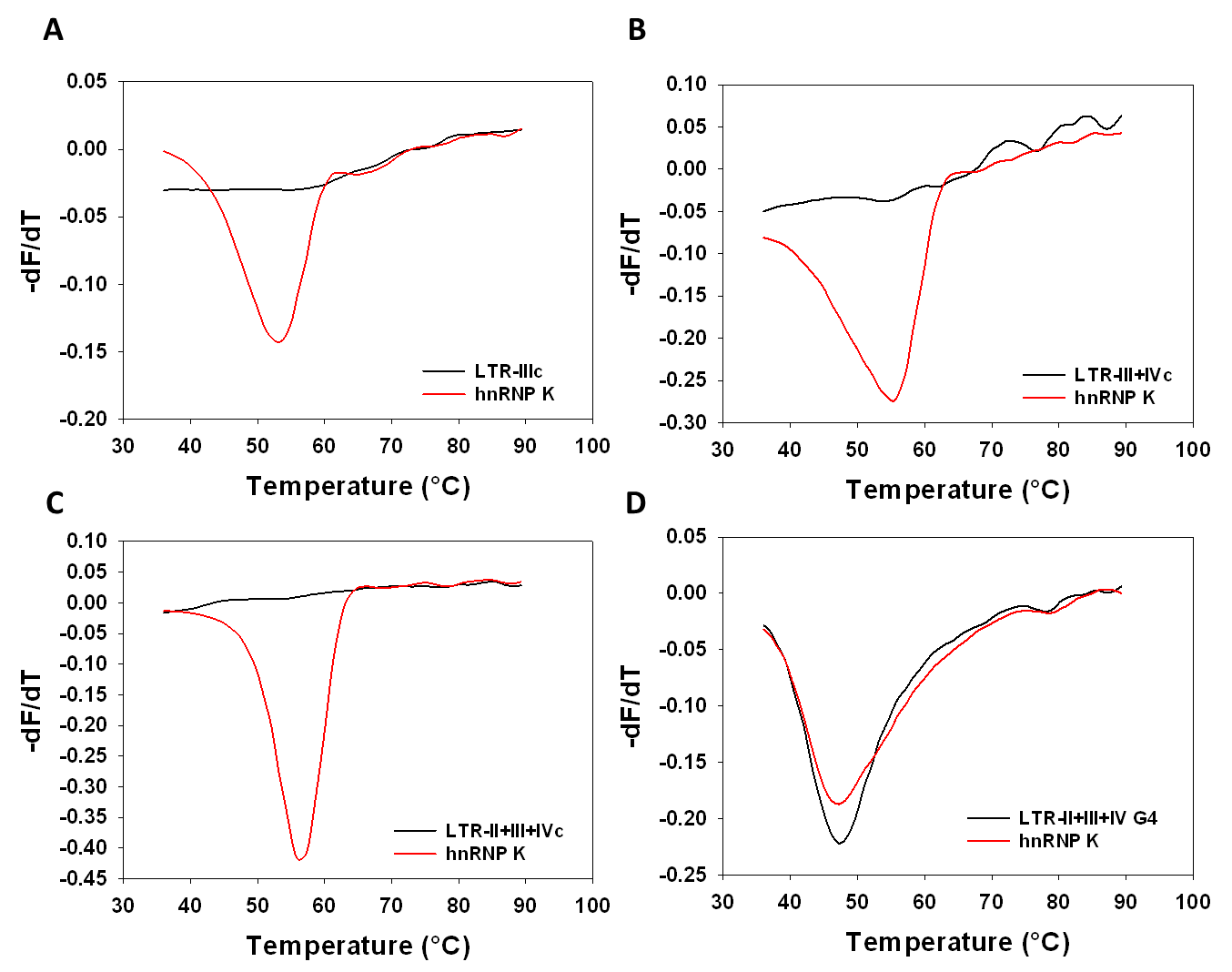


**Figure S10**. FRET-melting assay performed in lithium cacodylate buffer. First derivative curves of LTR-IIIc (A), LTR-III+IVc (B), LTR-II+III+IVc (C) and LTR-II+III+IV G4 (D) (100 nM) in the absence (black lines) and presence (red lines) of recombinant hnRNP K (500 ng).
